# EPIFBMC: A New Model for Enhancer–Promoter Interaction Prediction

**DOI:** 10.3390/ijms26168035

**Published:** 2025-08-20

**Authors:** Chengfeng Bao, Gang Wang, Guojun Sheng, Yu Chen

**Affiliations:** College of Computer and Control Engineering, Northeast Forestry University, Harbin 150040, China; baocf@nefu.edu.cn (C.B.); wanggang@nefu.edu.cn (G.W.); shengguojun@nefu.edu.cn (G.S.)

**Keywords:** gene expression, enhancer–promoter interactions, deep learning, genomic features, DNA sequence, 3C, Hi-C, ChIA-PET

## Abstract

Enhancer–promoter interactions (EPIs) play a key role in epigenetic regulation of gene expression, dominating cellular identity and functional diversity. Dissecting these interactions is crucial for understanding transcriptional regulatory networks and their significance in cell differentiation, development, and disease. Here, we propose a novel deep learning framework, EPIFBMC (Enhancer-Promoter Interaction prediction with FBMC network) that leverages DNA sequence and genomic features for accurate EPI prediction. The FBMC network consists of three key modules: the Four-Encoding module first encodes the DNA sequence in multiple dimensions to extract key sequence information; then the BESL (Balanced Ensemble Subset Learning) adopts an integrated subset learning strategy to optimize the feature-learning process of positive and negative samples; finally, the MCANet module completes the training of EPI prediction based on a Multi-channel Network. We evaluated EPIFBMC on three cell line datasets (HeLa, IMR90, and NHEK), and validated its generalizability across three independent datasets (K562, GM12878, HUVEC) through cross-cell-line experiments, comparing favorably with state-of-the-art methods. Notably, EPIFBMC balances genomic feature richness and computational complexity, significantly accelerating training speed. Ablation studies identified two key DNA sequence features—positional conservation and positional specificity score—which showed critical predictive value across a benchmark dataset of six diverse cell lines. The computational testing show that EPIFBMC shows excellent performance in the EPI prediction task, providing a powerful tool for decoding gene regulatory networks. It is believed that it will have important application prospects in developmental biology, disease mechanism research, and therapeutic target discovery.

## 1. Introduction

In the human genome, non-coding DNA accounts for about 98%, of which enhancers and promoters are two key elements in gene regulation. The interaction between them (EPIs) plays a vital role in gene expression regulation, cell differentiation and disease mechanisms [1]. For example, mutations in enhancers and promoters destroy the normal interaction between them, leading to abnormal gene expression, which in turn causes diseases such as β-thalassemia and congenital heart disease [2,3]. Therefore, the study of EPIs has important biological significance. In recent years, with the advancement of chromosome conformation capture technology, people have taken a step closer to the study of EPIs. For example, high-throughput chromosome conformation capture (Hi-C) [4], chromosome conformation capture (3C) [5], circular chromosome conformation capture (4C) [6], carbon copy chromosome conformation capture (5C) [7], and paired-end tag chromatin interaction analysis (ChIA-PET) [8]. Based on these experimental data, it was found that many enhancers regulate distant genes in the genome with the help of long-range EPIs; when the resolution of Hi-C reaches a high level, single EPIs can be measured, and the genomic characteristics of the enhancer’s true target gene can be distinguished from other nearby expressed genes. Although these technologies can detect enhancer-promoter interactions, the chromatin interaction mechanism at the genomic sequence level is still unclear, and it is still expensive, time-consuming and labor-intensive to obtain these data by significantly increasing the sequencing depth [9]. Therefore, there is an urgent need to build efficient computational methods to identify and study EPIs, and the experimental data generated by high-throughput sequencing technology has created conditions for this [10].

Subsequently, increasing evidence has shown that DNA sequence and epigenomic features are informative predictors of regulatory interactions [11], as they play a role in transcriptional regulation and chromatin folding by controlling DNA accessibility and recruitment of specific proteins [12,13]. Based on these features, many computational methods have been developed to study enhancer-promoter organization as an alternative to expensive experimental methods. For example, TargetFinder [14], A predictive modeling approach for cell line-specific long-range regulatory interactions(RIPPLE) [15], and Global view of enhancer–promoter interactome in human cells(IM-PET) [16] rely on multiple functional genomic features such as DNA methylation, transcription factor binding, and histone modifications. In addition, several EPIs prediction models based on deep learning networks have been proposed to improve performance. For example, Sequence-based Prediction of Enhancer–Promoter Interactions with Deep learning(SPEID) [17] uses convolutional neural networks to predict enhancer–promoter interactions based solely on sequence features; Enhancer–Promoter Interaction prediction using pre-trained DNA Vectors and Attention Network(EPIVAN) [18] extracts local and global sequence features through pre-trained DNA vectors, convolutional and recurrent neural networks, and attention mechanisms; Restricted Attention-based Enhancer–Promoter Interaction prediction (RAEPI) [19] introduces a restricted attention mechanism based on convolutional feature extraction to simulate enhancer–promoter interactions; Prediction of Enhancer–Promoter interactions (PEP) [12] uses two complementary strategies, motif and k-mer, to mine sequence information; Simple Convolutional Neural Network for EPI prediction (SIMCN) [20] uses a convolutional neural network with a simple structure to achieve prediction performance comparable to that of complex architectures. All of these methods use only DNA sequence data for prediction. Enhancer–Promoter Interaction prediction using Hybrid features and Communicative learning (EPIHC) [21] combines sequence features with genomic features and captures interaction information through a communicative learning module; Enhancer–Promoter Interaction prediction using Deep Learning and Matching Heuristic (EPI-DLMH) [22] introduces a matching heuristic method to enhance feature interaction modeling; and Enhancer–Promoter Interaction Prediction using a Pre-trained Deep Learning Framework (EPIPDLF) [23] uses a pre-trained deep learning framework and has interpretable analysis capabilities. Although some computational methods facilitate the prediction of EPIs, their reported performance is exaggerated due to overfitting caused by random splitting and high overlap of samples [13,24].

First, the use of genomic feature data can capture the specificity of cell types and provide more information. Based on this information, EPIs can be better predicted, but too much genomic feature data will make the calculation too complicated, so it is necessary to strike a balance between feature richness and computational complexity. Second, the sample data set has the disadvantages of imbalance and random segmentation. Although Mao et al. [25] proposed data augmentation tools, it is impossible to verify whether the generated samples are biologically meaningful, and random sampling easily leads to the loss of important negative sample information. Therefore, the performance of these models is not satisfactory. In addition, because the encoding method they use is relatively simple and the machine learning or deep learning-based models they build are relatively simple, the models cannot fully extract and learn the information features of gene sequences, and thus cannot make good predictions. To address the aforementioned issues, we propose the FBMC((F)Four-Encoding + (B)BESL + (MC)MCANet) model, which tackles them one by one through a combined scheme of ‘multi-feature encoding + sample balancing strategy + hybrid network architecture’.

With the rise of deep learning, sequence encoding methods have attracted much attention. In addition to one-hot encoding, several studies have put forward improvement ideas: Zhang [26] and Gong [27] used word2vec word embedding to improve performance; Lv [28] identified key features of chromatin loops through various encodings; Zhang et al. [29] abandoned traditional encoding schemes, and the prediction of circRNA-RBP binding sites [30] also adopted new encoding strategies. Moreover, although methods such as iLearnPlus [31] integrates multiple feature extraction techniques and machine learning algorithms to provide a flexible platform for biological sequence analysis, iPro-WAEL [32] introduces a weighted logistic regression strategy in the feature selection stage to enhance the effectiveness of feature utilization and Meta-mCpred [33] combines multi-layer feature representations with an ensemble learning framework to improve the robustness of prediction, they still face limitations in handling high-dimensional sequence information and complex regulatory relationships. Therefore, we propose a combination of multiple encoding schemes to further enhance the generalization ability and prediction accuracy of our model.

In summary, in order to solve the above problems, we designed a deep learning method. The main contributions of this study are as follows: (1) We propose a new and efficient model for predicting EPIs, EPIFBMC. We use three datasets to evaluate the advantages of this model over existing methods, and further validate it across cell lines on three other independent datasets to demonstrate its generalization ability. The computational testing shows that EPIFBMC has the best performance and good robustness in the EPI prediction task. (2) To solve the problem of dataset imbalance during training we use a new method—BESL. Experimental evaluation is performed through five-fold cross-validation, and the results show that this balancing strategy has a significant effect in improving the model’s prediction ability, demonstrating the importance of data balancing for EPI prediction models. (3) We analyze the performance differences between the two strategies of using only DNA sequence features and combining DNA sequence and genomic features, and strike a balance between the richness of genomic features and computational complexity to ensure the comprehensive optimization of the model in terms of prediction accuracy and training efficiency. (4) We identify two important DNA sequence features—position conservation and position specificity scores. In ablation experiments on a single benchmark dataset of six different cell lines, these two features significantly contribute to the identification of EPIs, further demonstrating the importance of DNA sequence features in the resolution of gene regulatory mechanisms.

## 2. Results

### 2.1. Performance Evaluation of Different DNA Sequence Features

**Figure 1:**
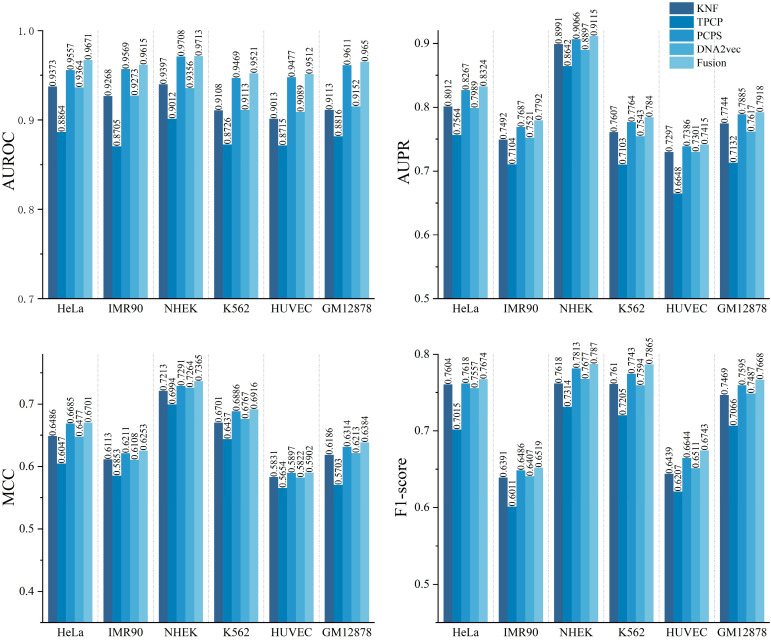
Performance evaluation of different DNA sequence features. This figure compares four DNA sequence feature encoding methods used in this study: KNF (K-mer Nucleotide Frequency): captures local sequence patterns by counting frequencies of nucleotide k-mers. TPCP (Trinucleotide Physicochemical Properties): combines trinucleotide frequencies with physicochemical property values. PCPS (Position Conservation and Position Special Scoring): measures sequence conservation and position-specific functional scores. DNA2vec: embeds DNA k-mers into continuous vector space using a word2vec-based model to capture semantic sequence information.

To verify the necessity of multi-feature fusion, we first evaluated the performance of single DNA sequence features (one-hot, KNF, TPCP, PCPS, DNA2vec). The results showed that the PCPS feature contributed the most significantly, and the performance was further improved after fusion(as shown in Figure 1). Due to limited chart size, we removed the weaker one-hot encoding to improve readability. One-hot encoding is the most basic sequence representation method, representing each base independently as a fixed-length sparse vector to preserve the most original sequence information. KNF (K-mer Nucleotide Frequency) captures local sequence patterns by calculating the frequency of k-mer nucleotides. TPCP (Trinucleotide Physicochemical Properties) combines trinucleotide frequencies with their physicochemical properties to characterize sequences. PCPS (Position Conservation and Position Special Scoring) captures core regulatory site information by measuring sequence conservation and position-specific functional scores. DNA2vec, based on word2vec, embeds DNA k-mers into a continuous vector space to extract context-dependent features. We have repeated these brief descriptions in the legend to Figure 1 to enhance the self-explanatory nature of the diagram. For six cell lines (HeLa, IMR90, NHEK, K562, HUVEC, and GM12878), we constructed models based on each feature respectively. Through five-fold cross-validation, we evaluated the performance using four metrics: AUROC, AUPR, MCC, and F1-score(see Section 4.3 for details). From the results (as shown in Figure 1), among all cell lines, the PCPS feature model performed the most prominently, with the average AUROC exceeding 0.95, which strongly demonstrates its ability to efficiently capture the conservation and position-specific information of E-P pairs. The KNF and DNA2vec feature models also showed good predictive ability, with the average values of each metric ranking second, indicating that nucleotide frequency and contextual information make certain contributions to EPI identification. Meanwhile, as a basic and commonly used sequence encoding method, one-hot encoding was included in the scope of single feature evaluation in this study. However, in actual tests, its overall performance across all cell lines was weaker than that of features such as PCPS, KNF, and DNA2vec, which indirectly highlights the value of exploring more targeted targeted sequence features and performing feature fusion. In addition, we constructed a model by fusing four features (KNF, TPCP, PCPS, DNA2vec) and found that the fusion strategy significantly improved the prediction performance. The fused model achieved the highest AUROC, AUPR, MCC, and F1-score across the six cell lines. This indicates that different DNA sequence features capture EPI patterns from multiple dimensions, and their combination can more comprehensively reveal sequence-driven regulatory mechanisms. It also confirms that the feature fusion strategy can effectively enhance the accuracy and generalization ability of EPI prediction in different cell types.

### 2.2. Performance Evaluation of Models Based Only on DNA Sequences

**Figure 2:**
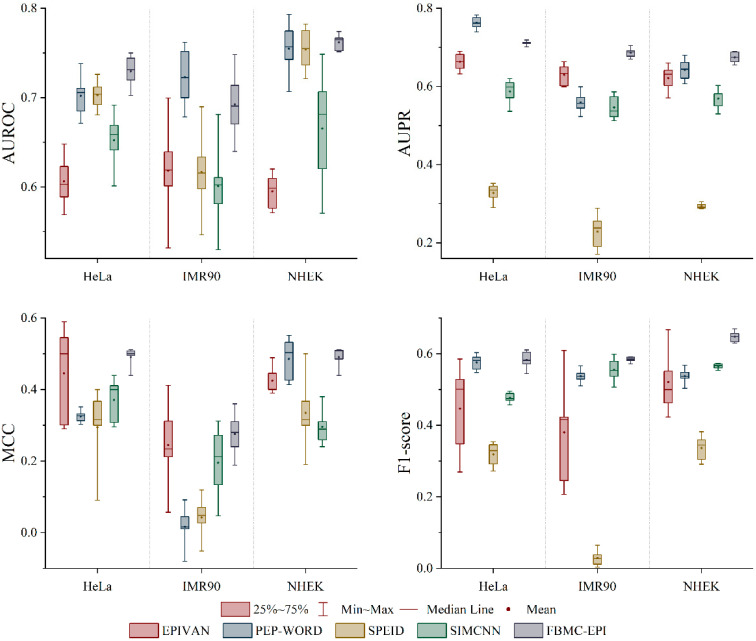
Performance comparison of DNA-only models on the experimental dataset, evaluated using AUROC, AUPR, MCC, and F1-score metrics across HeLa, IMR90, and NHEK cell lines. The box plots display the 25–75% interquartile range (box), minimum-maximum range (whiskers), median line, and mean value (red dot) for each model.

Based on the above feature analysis, we further constructed a prediction model that relies solely on DNA sequences. Compared with existing single encoding methods (such as SPEID and SIMCNN), the performance of the EPIFBMC model is optimal (as shown in Figure 2). In the research on EPIs, Zhang et al. [19], Yang et al. [12] and Zhuang et al. [20] researchers have also attempted to make predictions using only DNA sequence information. Although DNA sequence information provides basic genetic coding capabilities in these studies, a single sequence feature often struggles to fully capture the complexity of EPIs. Traditional one-hot encoding or k-mer statistics-based methods have information loss when characterizing sequence features, and it is difficult to effectively model the biophysical properties, evolutionary conservation, and contextual dependence of the sequence. Therefore, in recent years, deep learning methods that combine multiple sequence feature-encoding methods have gradually attracted attention to improve the performance and stability of EPI prediction. This study uses Four-Encoding as a sequence representation strategy and constructs a prediction model with multi-dimensional feature fusion. We compared the results with recent studies based only on DNA sequences. The experimental results of three experimental sets (as shown in Figure 1) show that compared with traditional one-hot or other single encoding methods, the Four-Encoding combined feature model we proposed has improved the AUROC, AUPR, MCC and F1-score evaluation indicators. For example, the average F1-score of the model on HeLa, IMR90 and NHEK is 0.1%, 0.4% and 0.9% higher than the second-ranked predictor, respectively. Among them, PCPS and DNA2vec combine evolutionary constraint information with contextual features, so that the model still has strong generalization ability under data imbalance, especially in the cross-cell line validation experiment below. It shows higher stability. In addition, we observed that multi-dimensional feature fusion can effectively alleviate the overfitting problem caused by data sparsity and sequence redundancy in EPI prediction tasks, and the calculation time of the model designed in this paper is lower than that of other models (Table 1). These results indicate that the sequence feature method based on the Four-Encoding combination can more fully explore the functional signals in the DNA sequence, thereby improving the robustness and biological interpretability of EPI prediction, and providing a new modeling idea for EPI prediction based only on DNA sequences.

### 2.3. Performance Comparison with State-of-the-Art Methods

**Figure 3:**
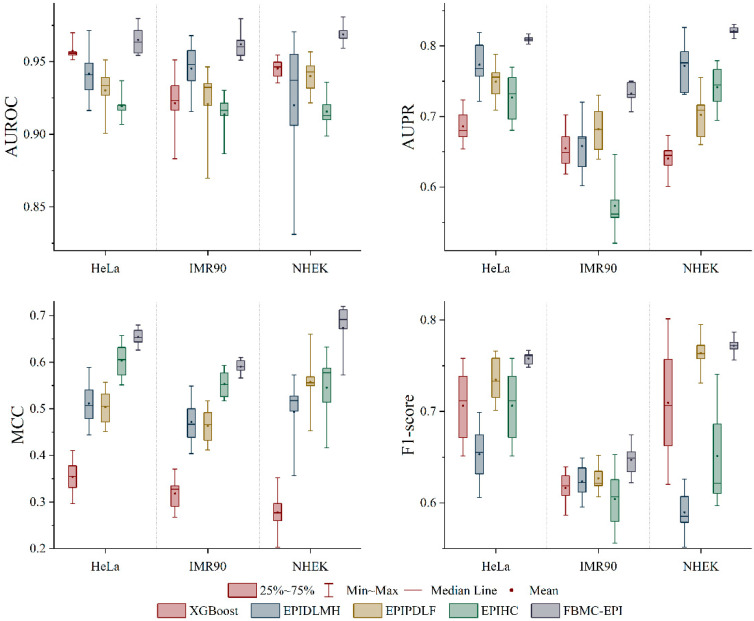
Model performance comparison using genomic and sequence features in three cell lines, evaluated using AUROC, AUPR, MCC, and F1-score metrics across HeLa, IMR90, and NHEK cell lines. The box plots display the 25–75% interquartile range (box), minimum-maximum range (whiskers), median line, and mean value (red dot) for each model.

On the basis of verifying the effectiveness using only DNA sequences, we constructed the complete EPIFBMC model by integrating genomic features (RAD21, ATAC-seq, etc.). Compared with existing mainstream methods (such as EPIPDLF and EPIHC), it achieves satisfactory results in metrics like AUROC and AUPR, with higher training efficiency (as shown in Figure 3 and Table 2). As can be seen from the figure, our model has a considerable advantage in the performance of the three cell lines (the average score values are all the highest), indicating that the performance of EPIFBMC is significantly better than the existing methods. Two main reasons may be that different encoding modes play different roles in the DNA sequence of enhancer–promoter, which can more comprehensively capture the complex information in the DNA sequence, and the combination of the genomic feature extraction module with attention mechanism and the effective DNA sequence module enables EPIFBMC to better extract potential information from the data. Specifically, the EPIFBMC model outperforms the second-ranked predictor by 0.8%, 1.2%, and 1.1% on HeLa, IMR90, and NHEK in the AUROC metric, respectively, which indicates that EPIFBMC has a higher ability to distinguish positive samples. Secondly, our model outperforms the second-ranked predictor by 0.4%, 0.9%, and 0.7% on HeLa, IMR90, and NHEK in AUPR performance, respectively, and the model improves most significantly on MCC, which increases by 1.1%, 0.9%, and 1.6%, respectively, indicating that the prediction results of our model are more correlated with the actual labels. Finally, the F1-score of the EPIFBMC model on HeLa, IMR90, and NHEK is 0.5%, 0.3%, and 0.1% higher, respectively, than the second-ranked predictor. To verify the statistical significance of the performance difference between EPIFBMC and the suboptimal model EPIPDLF, we integrated the data of corresponding indicators from three cell lines (IMR90, NHEK, and HeLa) for analysis. Using Python3.8’s scipy (version 1.5.4) library, we conducted paired t-tests on the four indicators (AUROC, AUPR, MCC, and F1) between EPIFBMC and the suboptimal model EPIPDLF. The results showed that for all indicators, the performance differences between EPIFBMC and EPIPDLF all satisfied *p* < 0.05 (AUROC: *p* = 0.022; AUPR: *p* = 0.036; MCC: *p* = 0.001; F1: *p* = 0.006), indicating that the performance improvement of EPIFBMC is statistically significant.

In summary, the EPIFBMC model robustly outperforms the state-of-the-art predictors in predicting EPIs. The reason why EPIFBMC performs well is that, on the one hand, our new data balancing method, BESL, enables the model to learn the features of positive and negative samples in a balanced way, which improves the prediction of EPIs; on the other hand, it is because our encoded DNA sequence features are more informative than other models. Fusion features can effectively improve the expressiveness of features, and are here combined with the attention mechanism to capture the dependencies and important features between different genomic features, so EPIFBMC has more comprehensive prediction capabilities. In addition, we further evaluated the difference in computational efficiency between this research method and existing models. In the experiment, all models were trained on a V100-32 GB GPU, and their running time was counted. The results are shown in Table 2. The total training time in HeLa, IMR90, and NHEK of XGBoost is about 54,620 s, EPIPDLF is the highest, reaching 56,841 s, and EPIHC is about 37,923 s, while this research method only takes 30,359 s, which greatly shortens the calculation time. This advantage is mainly attributed to the balance we have achieved between feature richness and computational complexity, which significantly reduces the number of parameters required for training. Overall, this research method improves computational efficiency while ensuring performance, which is more advantageous than existing methods.

### 2.4. Cross-Cell-Line Validation

**Figure 4:**
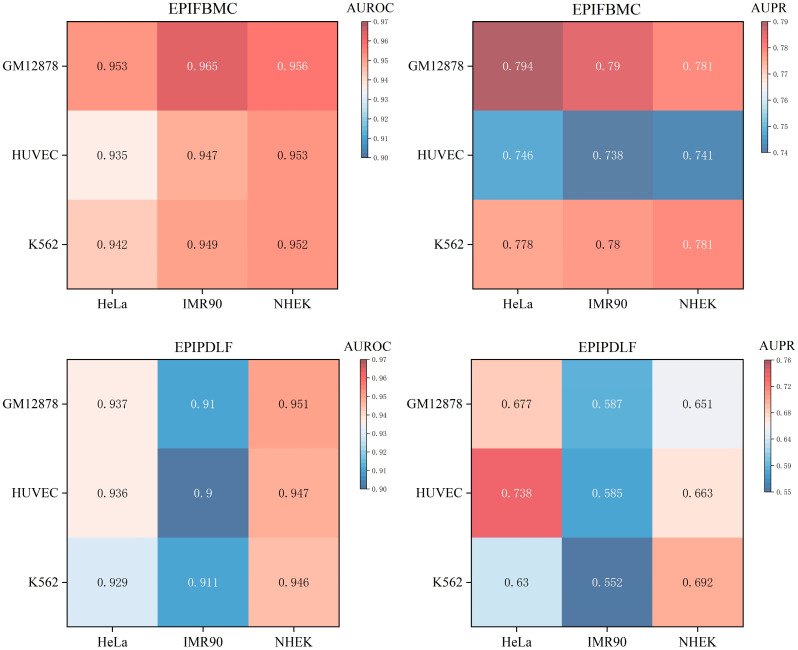
AUROC and AUPR comparison of EPIFBMC vs. EPIPDLF across cell lines. The heatmaps display the AUROC (**left**) and AUPR (**right**) performance of EPIFBMC (**top**) and EPIPDLF (**bottom**) on GM12878, HUVEC, K562, HeLa, IMR90, and NHEK cell lines. Color intensity indicates the magnitude of the performance metric, with darker colors representing higher values for AUROC and AUPR.

To test whether the model captures universally applicable EPI rules, we performed cross-cell-line validation. The results showed that EPIFBMC still outperformed EPIPDLF in blood-related cell lines (such as K562) (as shown in Figure 4), demonstrating that it is not dependent on specific cellular environments and possesses robust generalization ability. Since EPIs exhibit significant specificity across different cell types [34], we used a heatmap to visually display the generalization ability of our model across various cell lines. In order to make the experimental conclusions more rigorous and reliable, we use independent datasets (K562, GM12878, HUVEC) for testing. The difference between these cell lines and the above HeLa, IMR90 and NHEK (non-blood-related) is that they are blood-related. If the AUROC and F1-score indicators on the independent test set are still high, it means that the model can effectively identify universally applicable EPI rules rather than relying on specific cell environments. As can be observed from Figure 4, although the model is only trained on non-blood-related cell lines (HeLa, IMR90, NHEK), it still performs better than the most advanced model EPIPDLF on blood-related test cell lines (K562, GM12878, HUVEC). Specifically, we found that the AUROC on K562, GM12878, and HUVEC was improved by 0.1%, 1.4%, and 0.6%, respectively, compared with the current state-of-the-art models, and the improvement in the AUPR indicator was more obvious. Overall, the prediction performance differences between different cell lines were small, and the heat map showed a relatively uniform high-scoring area, indicating that the model can effectively learn the EPI rules across cell types and has good generalization ability. In addition, no obvious skewness was observed in the heat map (i.e., the model performed well on some cell lines and performed extremely poorly on other cell lines), which further verified the stability of the model. In summary, the experimental results prove that the model combining KNF, TPCP, PCPS, and DNA2vec multidimensional sequence features can effectively extract key information of enhancer–promoter interactions and show strong generalization ability in cross-cell line tasks.

### 2.5. Analysis of Loss Performance for Training and Testing Datasets

**Figure 5:**
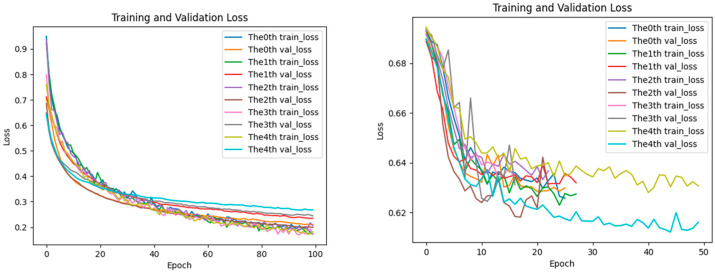
HeLa loss of performance on training and test datasets (**left**: EPIFBMC, **right**: EPIPDLF).

**Figure 6:**
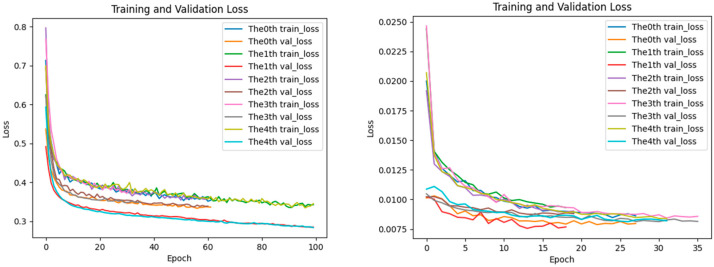
IMR90 loss of performance on training and test datasets (**left**: EPIFBMC, **right**: EPIPDLF).

**Figure 7:**
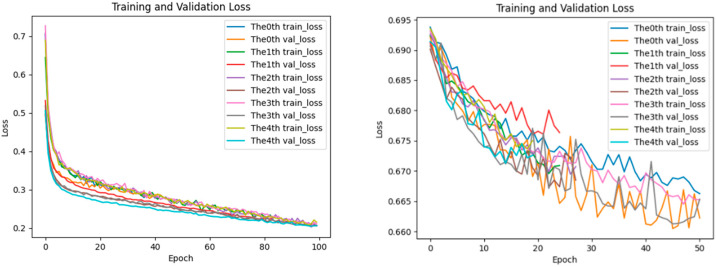
NHEK loss of performance on training and test datasets (**left**: EPIFBMC, **right**: EPIPDLF). Because the X-axis scale ranges on the left and right models are different, we used a fixed-point analysis method. For example, at Epochs 10, 20, and 30 of the HeLa cell line, the loss of EPIFBMC decreased rapidly and stabilized, while the loss of EPIPDLF decreased more slowly and the final loss value was higher than that of EPIFBMC. These figures show the variation trends of training loss (train loss) and validation loss (val loss) of the EPIFBMC and EPIPDLF models with the number of training epochs during the training process on the HeLa, IMR90, NHEK cell line dataset.

The aforementioned performance advantages can be further explained by the training process: the loss curve of EPIFBMC declines more rapidly, and the gap between the training loss and validation loss is smaller (as shown in Figure 5, Figure 6 and Figure 7). This indicates that it has better convergence efficiency and anti-overfitting ability, providing technical support for its high performance. In the statistics of the loss function changes of the EPIFBMC model, we show the performance of the model on the training set and test set. We use categorical crossentropy as the loss function to evaluate its convergence and generalization ability. In order to understand the performance of EPIFBMC more comprehensively, this paper compares it with the current optimal model EPIPDLF, focusing on the training process of the model, the change of the loss function curve, and the generalization ability of the final model. From the perspective of the training process, EPIFBMC combines positive and negative samples and balances them through innovative feature coding methods and sample processing strategies, thus solving the problem of data imbalance while maintaining efficient training, and improving the training efficiency, so that the model can learn from positive samples more effectively. EPIPDLF uses another training strategy, which treats DNA sequences as text data and transforms them into “Biological vocabulary” by constructing a vocabulary, extracting features using CNN, GRU, etc.; the prediction performance is improved by combining pre-training, adversarial learning strategy and genomic information fusion, but the framework has certain defects. Firstly, there is no data balance in data processing, and while the prediction performance is improved by using the proposed framework, it may affect the recognition ability of the model for minority samples. Secondly, it fails to make full use of all the features of DNA sequences, and there are some limitations in feature mining and utilization. In terms of the change in the loss function and generalization ability, both the training loss curve and the validation loss curve of EPIFBMC show a rapid downward trend at the initial stage (Figure 5, Figure 6 and Figure 7), and the loss gradually stabilizes as the epoch increases. In EPIPDLF, although the loss curve shows a downward trend, the performance of the loss curve of the validation set and the training set fluctuates on the data set. In addition, by observing the correlation between EPIFBMC training loss and validation loss, it is found that the gap between the two is not obvious, indicating that the model is more stable in the three cell lines and its generalization ability is stronger than that of EPIPDLF.

Besides, EPIFBMC has significant advantages in convergence speed and training efficiency. In the analysis of the loss curve, the loss value of the EPIFBMC decreases rapidly at the beginning of training, showing that the model can quickly learn important features from the data. In contrast, the training process of EPIPDLF is relatively long and complex, and the convergence speed is slow due to the use of CNN, GRU and other extracted features, although GRU can deal with the long-distance dependence of sequences. On large-scale datasets, EPIFBMC achieves more efficient feature extraction and fast convergence by combining convolutional neural network (CNN) and multi-attention mechanisms, using the combination of local features and global features. This makes the EPIFBMC reach the optimal model faster than the EPIPDLF under the same training conditions. In summary, EPIFBMC comprehensively surpasses EPIPDLF in terms of model performance, training efficiency and generalization ability, and the model shows good prediction effect and robustness through more efficient training process and strong learning ability.

## 3. Discussion

In this study, we proposed a novel and efficient enhancer–promoter interaction (EPI) prediction model, EPIFBMC, which showed superior performance and good generalization ability in experiments on multiple benchmark datasets and independent cell lines by integrating multiple sequence feature encodings, innovative sample balancing strategies, and combining multi-layer convolution and multi-channel attention convolutional networks (MCANet). The BESL strategy we designed effectively alleviated the model bias problem caused by the imbalance of positive and negative samples, and significantly improved the robustness and prediction accuracy of the model. While EPIFBMC achieves improved performance, our operational efficiency has also been enhanced. The average training time of EPIFBMC has been shortened by approximately 42%. This efficiency improvement is mainly attributed to the optimization of the BESL balancing algorithm, four-encoding method, and a small number of genomic features, which have significantly reduced the running time. Through comparative analysis of different feature combination strategies, we further clarified the importance of the synergistic fusion of DNA sequence and genomic features for performance improvement, and identified two key sequence features with significant prediction contributions, namely position conservation and position-specific scores. In summary, EPIFBMC provides a reliable deep learning framework for the efficient identification of EPIs, and also provides new research ideas and technical support for exploring gene regulatory mechanisms.

Despite the excellent performance of EPIFBMC, there are still several aspects that can be further improved in the future. First, the potential role of sequence information may not be fully revealed. Therefore, under the premise of effectively reducing noise interference, in-depth mining of this implicit information is expected to become a key strategy to improve prediction accuracy. Secondly, since the imbalance of data samples is common in EPI research, it is also a worthy research direction to adopt reasonable technologies (such as generative adversarial networks (GAN)) to expand the positive data set in the future. In addition, the integration of functional annotation and experimental verification is also the key to promote the EPI prediction method from theory to application. By combining known functional databases (such as ENCODE (https://www.encodeproject.org/), FANTOM5 (https://fantom.gsc.riken.jp/5/)) and CRISPR experimental data, functional annotation and verification of model prediction results will help improve the credibility and practicality of the results. These directions will provide a more robust and comprehensive technical foundation for EPI research.

## 4. Materials and Methods

### 4.1. Benchmark Datasets

**Figure 8:**
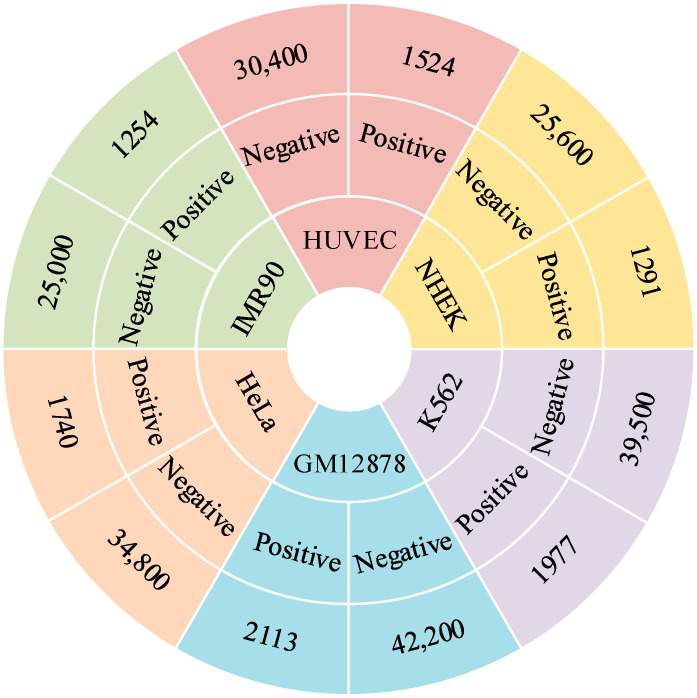
Single benchmark dataset for six cell lines. This circular chart shows the distribution of positive and negative samples in the single benchmark dataset for six cell lines, namely GM12878, HeLa, IMR90, HUVEC, NHEK, and K562. Different colored sectors in the chart represent different cell lines, and the numbers within the sectors indicate the quantity of the corresponding sample type (positive or negative).

To evaluate the effectiveness of the EPIFBMC model, we used a benchmark dataset from Target Finder [14], which includes EPI data of six different human cell lines (as shown in Figure 8), namely K562 (leukemia-derived mesenchymal cells), IMR90 (fetal lung fibroblasts), GM12878 (lymphoblasts), HeLa-S3 (cervical cancer cells), NHEK (epidermal keratinocytes), and HUVEC (umbilical vein endothelial cells). Hi-C technology was used to perform high-precision whole-genome measurements on each cell line, thereby dividing EP pairs into interacting (positive samples) and non-interacting (negative samples) categories, and the ratio of positive to negative samples in each cell line was 1:20. In order to verify whether this model has the ability to generalize to cell lines from different sources, we divided the benchmark dataset into two groups: non-blood-related (HeLa, NHEK, IMR90) and blood-related (K562, GM12878, HUVEC). The first group was used as the experimental set, and the remaining three datasets in the second group were used as independent data for cross-cell line validation. In addition, due to limited computing resources, we only introduced four genomic features from the GATv2EPI [35] study, namely transcription factor binding (RAD21), chromatin accessibility (ATAC-seq), histone modification (H3K27ac) and logarithmic distance (Distance) between EPs. These data were obtained from epigenomic data files in BigWig format downloaded from the ENCODE and NIH Roadmap Epigenomics projects. These files contain the signal value of each base pair in the whole genome, which can directly reflect the chromatin state and transcription factor binding. For RAD21 data, ChIP-seq (chromatin immunoprecipitation sequencing) uses specific antibodies to capture RAD21-bound DNA fragments and performs high-throughput sequencing to obtain the binding sites of RAD21 on the genome, which are represented by peak signal intensity. For ATAC-seq and H3K27ac data, the original experimental measurements are stored in BED format, and each BED file represents a small range of genomic sub-regions and their experimentally measured signal values. In order to convert BED files to BigWig format for numerical calculations, Bedtools and bedGraphToBigWig tools are used for merging and conversion to ensure a one-to-one correspondence between each base pair and the signal value.

### 4.2. Model Framework

#### 4.2.1. Overall Framework

**Figure 9:**
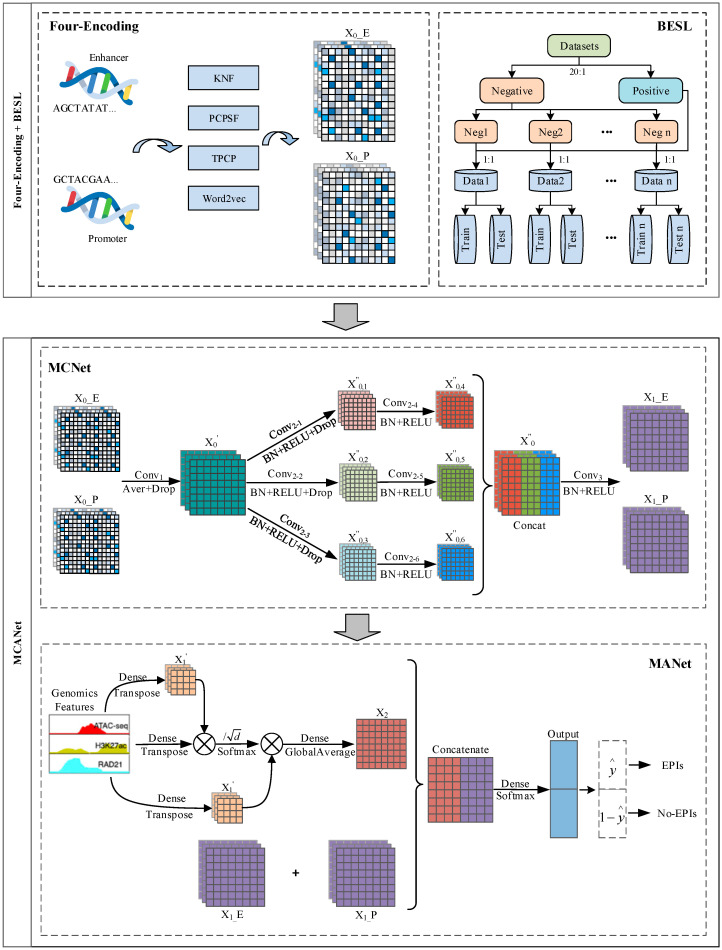
Overall framework diagram of EPIFBMC, which consists of Four-Encoding, BESL, MCANet, and output. The Four-Encoding part performs four encoding processes of KNF, PCPSF, TPCP, and Word2vec on the DNA sequences of Enhancer and Promoter to generate corresponding feature matrices; the BESL part processes positive and negative samples to solve the problem of data imbalance; the MCANet part further processes the features through a series of operations such as convolution, batch normalization, and activation functions; finally, the output module obtains the prediction results regarding enhancer-promoter interactions (EPIs) and non-interactions (No-EPIs).

The overall framework of EPIFBMC is shown in Figure 9. It includes the Four-Encoding module for gene sequences, the BESL balanced dataset module, the MCANet module, and the output module. The functions of each module are as follows:

Four-Encoding: We use four sequence encoding schemes to encode EP pairs, corresponding to four different sequence features, including KNF (k-mer nucleotide frequency), TPCP (trinucleotide physicochemical properties), PCPS (position conservation and position specificity score) and DNA2vec (sequence context feature). These four encodings are merged in the last dimension to obtain the final feature vector. These encoding schemes will be described in detail in the following sections.

BESL: A new method called Balanced Ensemble Subset Learning (BESL) to solve the model bias problem caused by data imbalance (1:20). This method divides negative samples into 20 subsets, and builds balanced sub-models (1:1) with all positive samples respectively. The performance is improved by integrating the average prediction results of the 20 sub-models, and combined with five-fold cross-validation and early stopping mechanism to ensure generalization ability and computational efficiency.

MCANet: This network is mainly composed of multiple convolutions and attentions, where multi-layer convolution operations are used to capture local patterns and structural information in the sequence, automatically learning and extracting important features in the data based on traditional CNNs. The attention sub-block focuses on the dependencies between genomic features, and captures the dependencies and important features between different features through the combination of multiple attentions.

Output: This sub-block merges sequence features and genomic features, and performs full connection layer and classification layer processing to obtain the final classification result.

#### 4.2.2. Four-Encoding

##### KNF (K-Mer Nucleotide Frequency)

In this study, we proposed KNF as a feature descriptor based on K-mer Encoding [36] to reflect short-range sequence order effects. In contrast to the traditional one-hot encoding method, k-mer patterns can integrate various short-range or local context information [37]. The method captures local patterns and short-range sequence features in sequences by counting the frequency of k-tuple nucleotide combinations. In KNF, each K-mer is converted into a vector of a specific size according to its length. The size of the vector is determined by $4^{K}$, where K is the number of nucleotides, encoded as vectors containing 4, 16, 64, and 256 elements, respectively, to generate a nucleotide frequency matrix $M_{K}$, with a size of $(L-K+1)\times 4^{K}$, where each row represents the frequency distribution of a K-mer in the following sequence:

(1) $$M_{K}=\left[e_{i_{1}}{\;e}_{i_{2}}\;\ldots {\;e}_{i_{L-K+1}}\right]$$

where $e_{i}$ is a vector of $4^{K}$ dimension, where only the ith position is 1 and the rest of the positions are 0. The frequency pattern matrices of different K values (K = 1, 2, 3, 4) are concatenated:

(2) $$M=\left\{M^{1},M^{2},M^{3},M^{4}\right\}$$

In this way, 340 feature vectors can be converted for each nucleotide.

##### TPCP (Trinucleotide Physicochemical Properties)

TPCP encodes the sequence by combining the frequency and physicochemical properties (such as hydrogen bonding, hydrophobicity, etc.) of trinucleotides. This encoding has been successfully applied to the prediction of DNA N4 methylcytosine sites [38]. Among them, physicochemical properties such as bending, rigidity, and DNase I sensitivity will affect chromatin structure and the binding of transcription factors, thereby affecting EPIs. First, the frequency of occurrence of all trinucleotides (AAA, AAC, AAG, …, TTT) in the sequence was counted. We used 11 physicochemical properties *pc*_1_–*pc*_11_. The values of these physicochemical properties were normalized before calculation. Then, the feature vector of each trinucleotide was determined by the product of its frequency and the physicochemical property value.

(3) $$V_{TPCP}=\{pc_{1}\times f_{AAA},\ldots ,pc_{1}\times f_{TTT},\ldots ,pc_{11}\times f_{TTT}\}$$

where $pc_{i}$ represents the ith physicochemical property of the trinucleotide, $f_{NNN}$ represents the frequency of occurrence of a trinucleotide, so the final generated vector feature dimension is 64 × 11 = 704.

##### PCPS (Position Conservation and Position Special Scoring)

Position conservation and position-specific scores can identify conserved sites and functional sites in sequences. These sites are the core regulatory regions of enhancers or promoters [28] and affect the formation of EPIs. The conservation expression of a nucleotide fragment of k length at the ith position is as follows. In this study, k is set to 6:

(4) $$C_{k}(i)=\sum \frac{{\left[f_{i}(n)-f_{e}\right]}^{2}}{f_{e}}$$

where $C_{k}(i)$ represents the conservation value of the nucleotide at the ith position. The larger the value, the higher the conservation. $f_{i}(n)$ represents the frequency of the nucleotide fragment n at the ith position. The background frequency $f_{e}$ is set to $1{/4}^{k}$. The results show that the conservation of the 6-tuple fragment is high at 10 sites (−12, −10, −14, −15, −16, −13, −37, −11, −36 and −2 sites).

The position score function is based on the generation of features of conserved sites and position-related score matrix. The dimension of the position-related score matrix is 4096 × 10. The two expressions are as follows:

(5) $$m_{xi}=\frac{n_{xi}}{N_{i}+\sqrt{N_{i}}}+\frac{f_{0}\sqrt{N_{i}}}{N_{i}+\sqrt{N_{i}}},\;PSF=\left\{\ln\left(\frac{m_{x1}}{f_{0}}\right),\ln\left(\frac{m_{x2}}{f_{0}}\right),\ldots ,\ln\left(\frac{m_{xn}}{f_{0}}\right)\right\}$$

where $n_{xi}$ and $f_{0}\sqrt{N_{i}}$ represent the true count and pseudo count of the nucleotide x at the ith position, $N_{i}$ and $\sqrt{N_{i}}$ represent the actual total count and total pseudo count at the ith position. The background frequency $f_{0}$ is set to 1/200.

##### DNA2vec (DNA Sequence Embedding)

This feature encoding is based on the word2vec word embedding model. Word2Vec is an algorithm for natural language processing and has been widely used in biological sequences in recent years [35,37]. It captures the semantic information of the sequence by learning the vector representation of the words (nucleotides) in the sequence. It actually uses the shallow two-layer neural network in word2vec for training. In this study, we divide the DNA sequence into “words” to solve the similarity problem in different sequences of k-mer features. For example, a DNA sequence is “AGGTCCA”. We take k = 5 consecutive nucleotides as a “word”, then the sequence has a total of 3 “words”: AGGTC, GGTCC, GTCCA. Therefore, if the length of a sequence is L, take k consecutive nucleotides as a “word”, then there are a total of L−k+1 “words”. If each DNA “word” is embedded as a feature vector of d dimension, the feature dimension of each sequence is $d\times \left(L-k+1\right)$. When the length of the sequences in the dataset is inconsistent, resulting in different feature dimensions, these features cannot be directly used for training deep learning models. To address this challenge, we use an adaptive pooling layer to unify the feature dimensions of each sequence. In this study, each “word” consists of nucleotides of length 5 and is mapped to an 8-dimensional feature space. Considering that the central tendency of the number of words in human EP sequences is about 1000, we set the output dimension of the adaptive pooling layer to 1000 × 8, which is the feature dimension of DNA2vec encoding.

Overall, the four encodings contribute differently: KNF provides basic nucleotide frequency information, which plays an important role in helping the model capture the distribution patterns of bases in the sequence; TPCP introduces a new feature dimension to the model from the perspective of the physicochemical properties of nucleotides; PCPS helps the model identify conservative patterns in key regions of the sequence by calculating positional conservation; DNA2vec uses pretrained word vectors to represent the contextual semantics of the sequence and performs prominently in capturing the semantic information of the sequence.

#### 4.2.3. BESL (Balanced Ensemble Subset Learning)

In this study, since the ratio of positive and negative samples in each cell line is 1:20, the imbalance of the data set will cause the model to be biased towards the majority class and unable to fully learn the characteristics of positive samples, resulting in low recall and F1-scores. However, traditional solutions have shortcomings. We adopted a new solution, BESL, to solve the data imbalance problem and use five-fold cross-validation to improve the generalization ability and reliability of the model. For any cell line, we first split all data into positive and negative samples, and divide the negative samples into 20 subsets according to the positive and negative sample ratio. Then, each subset and all positive samples are constructed into 20 sub-models, so that the data set in each sub-model is 1:1 balanced, and 80% of them are used as training sets and validation sets, and 20% are used as test sets. In order to prevent the computational complexity of the 20 sub-models from being too high, when the loss on the validation set has not improved in five consecutive epochs, we use this as an early stopping mechanism. Secondly, these sub-models will produce their own training processes and prediction results. We average these 20 prediction results to obtain the final prediction results. The specific steps are as follows: (i) count the ratio of positive and negative samples in the training set; (ii) divide the negative samples into ratio-folds, and select the corresponding negative samples in each fold to integrate a new sub-model with the positive samples; (iii) the new training set is further divided into a training subset and a validation subset. The model is trained with the configuration of an early stopping strategy to prevent overfitting, where the monitored metric is set as the validation loss. Training will automatically stop when the validation loss fails to decrease (or even increases) for five consecutive epochs.; (iv) predict the test set using the trained model and averaging the predictions of the RATIO folds; (v) using a five-fold cross validation approach and taking the above actions for each fold.

#### 4.2.4. MCANet-Model

##### MCNet (Multi-Scale Convolutional Network)

After the above encoding, to achieve feature extraction and context learning of DNA sequence information, we designed MCNet. Although traditional CNN can capture local sequence patterns, it has limitations in processing high-dimensional complex data. Therefore, MCNet gradually extracts and processes input sequence features through a combination of convolution layers, pooling layers, normalization layers, activation functions, and Dropout layers to achieve effective feature extraction of EPIs. This module consists of a starting and ending convolution unit, as well as three parallel convolution sub-units. The convolution kernel of the starting convolution unit is set to 9, using 64 filters, and the size of the feature map is reduced through an average pooling operation, with the expression as follows:

(6) $$x_{0}\prime =Drop\left(AvePolling1D\left(Con1D\left(x_{0},‘relu’\right)\right)\right)$$

Then it is divided into three parallel convolution sub-units. Each sub-unit has the same structure and is independent of the others. They are composed of two convolution layers, with convolution kernel sizes of five and three, respectively, using 32 and 64 filters, and using two-batch normalization BN and RELU for activation:

(7) x0,i′=ReLUBatNormCon1DDropReLUBatNormCon1Dx0′

The output feature maps of the three parallel convolution sub-blocks are merged in the channel dimension to form a new feature map, and the feature map is Dropout twice:

(8) x1=DropReLUBatNormDropCon1Dconcatx0,1′,x0,2′,x0,3′

##### MANet (Multi Attention Network)

For the four genomic features (RAD21, ATAC-seq, H3K27ac, and Distance), MANet is introduced into MCANet, which incorporates multiple attention mechanisms to extract key feature dependencies. First, Min-Max normalization and linear transformation are applied to obtain an input matrix suitable for Transformer. Then, attention scores are calculated through dot product (including masking to suppress invalid positions), and attention weights with a sum of 1 are obtained through normalization. Finally, the output is generated by weighted summation. This mechanism can flexibly identify feature correlations and improve the ability to recognize EPIs. The specific implementation steps are as follows:

(9) $$x=\left[RAD21,ATAC-seq,H3K27ac,Distance\right]$$

The four genomic features are combined and projected using linear transformation (Fully Connected layer) to form a d dimensional feature vector, which can be represented as a matrix F, with each row corresponding to a different feature category: $F\in R^{d\times 4}$.

(10) $$u^{h}=\tanh\left(xW^{h}+b^{h}\right)$$

Here, $W^{h}$ and $b^{h}$ are the weight matrices and biases of the hth attention. The intermediate representation of the input is calculated by linear transformation (dot product and addition) and nonlinear activation function (tanh). Multiply it with the weight vector $w^{h}$ of attention to get the hth attention score of the current element, and use the exponential function to convert the attention score into a probability form to normalize it across all inputs, calculated as follows:

(11) $$a^{h}=\frac{\exp\left(u^{h}w^{h}\right)}{\sum_{i=1}^{T}\exp\left(u^{h}w^{h}\right)+\varepsilon }$$

By summing all weighted inputs, the output of the hth attention is obtained, and all outputs are merged together to calculate the final output:

(12) $$output=concat\left(att_{1},att_{2},\ldots ,att_{h}\right)$$

To reduce the high dimensionality of the output features from MANet, subsequent steps sequentially involve global average pooling for dimensionality reduction, followed by the application of batch normalization and a Dropout layer to enhance training efficiency and generalization ability. Then, a fully connected layer is used to map the features to 128 dimensions, which are concatenated with the enhancer–promoter DNA sequence feature vectors processed by MCNet. Finally, the prediction of E–P interactions is made through a fully connected layer and a softmax classification layer.

### 4.3. Effect Evaluation Indicators

This study performed five-fold cross validation on six cell line datasets [36]. Since ACC cannot capture the performance of an imbalanced ratio of positive and negative samples, this paper abandoned ACC [22,23] and used the following four evaluation indicators: area under the ROC curve (AUROC) [22,23], area under the PR curve (AUPR) [22,23], Matthews correlation coefficient (MCC) [22,23] and F1 score [22,23] to comprehensively evaluate the predictive performance of the model [39]. In this experiment, the indicator value is positively correlated with the performance. The following are the definitions of these indicators.

### 4.4. Experimental Environment

All deep learning-based model training and feature extraction tasks were conducted in a high-performance computing environment equipped with a Tesla V100 GPU (32GB video memory), 4-core CPU, 32 GB RAM, and 100GB disk storage, which supported efficient data loading and intermediate result storage during model iterations.

Other tasks, including data preprocessing (e.g., sequence encoding and dataset splitting), statistical analysis of experimental results, and visualization of figures, were performed on a personal computing setup with an AMD Ryzen 7 5800H with Radeon Graphics (base 3.20 GHz), 16.0 GB RAM (13.9 GB available), and x86_64 architecture.

## 5. Conclusions

Overall, FBMC-EPI demonstrates excellent performance in prediction accuracy, training efficiency, and cross-cell line generalization, proving its value as an effective and stable tool for enhancer-promoter interaction (EPI) prediction. By integrating multiple sequence encodings with key genomic features, the model improves prediction accuracy and reveals the important synergistic role of DNA sequence features alongside positional conservation and specificity scores in gene regulation.

Notably, differences in prediction results reflect the complexity of enhancer-promoter interactions, which depend not only on DNA sequence but also on cell type–specific chromatin states and three-dimensional genome architecture. Although the precise mechanisms remain unclear, these findings highlight the multilayered nature of regulatory specificity, offering important insights for a deeper understanding of gene expression regulation.

In the future, integrating functional genomic data and experimental validations—such as CRISPR-based assays and Hi-C data—will enhance the biological interpretability of the model. Additionally, addressing sample imbalance by employing techniques like generative adversarial networks (GANs) or self-supervised learning to augment positive datasets represents an effective path to further improve model performance. FBMC-EPI provides a solid foundation for EPI prediction and gene regulation research, while also guiding future optimization and practical applications.

## Figures and Tables

**Table 1:** Average running time (seconds) of different models on benchmark datasets.

Model/Cell Line	HeLa	IMR90	NHEK
EPIFBMC	5563	5614	1406
SIMCNN	16,615	12,156	12,038
SPEID	24,137	20,407	20,476
PEP-WORD	21,034	18,374	17,512
EPIVAN	16,128	12,467	12,168

**Table 2:** Average running time (seconds) of different models on benchmark datasets.

Model/Cell Line	HeLa	IMR90	NHEK
EPIFBMC	12,944	9101	4314
EPIPDLF	21,417	18,179	17,245
EPIHC	15,481	12,067	10,375
EPIDLMH	20,131	17,647	16,842
XGBoost	19,522	18,822	16,379

## Data Availability

The source code and dataset for this study have been uploaded to https://github.com/Fated-2/EPIFBMC (accessed on 20 May 2025).

## Abbreviations

The following abbreviations are used in this manuscript:

EPIs	Enhancer–Promoter Interactions
BESL	Balanced Ensemble Subset Learning
Hi-C	high-throughput chromosome conformation capture
3C	chromosome conformation capture
4C	circular chromosome conformation capture
ChIA-PET	paired-end tag chromatin interaction analysis
